# circRNA expression pattern and ceRNA network in the pathogenesis of aseptic loosening after total hip arthroplasty

**DOI:** 10.7150/ijms.48014

**Published:** 2021-01-01

**Authors:** Shenghui Ni, Tianlong Jiang, Shimin Hao, Peng Luo, Penghao Wang, Yaser Almatari, Yu Wang, Zhiyu Zhang, Lei Guo

**Affiliations:** 1 Department of Orthopedic Surgery, First Affiliated Hospital, China Medical University, Shenyang, Liaoning, 110001, P.R. China; 2 Department of Orthopedic Surgery, Fourth Affiliated Hospital, China Medical University, Shenyang, Liaoning, 110001, P.R. China

**Keywords:** high-throughput sequencing, competitive endogenous RNA, circular RNA, total hip arthroplasty, osteolysis

## Abstract

Increasing evidence has demonstrated that circular RNA (circRNA) exerts important function in the pathogenesis of some diseases. While, the contributions of circRNAs to aseptic loosening after total hip arthroplasty (THA) remain largely unknown. Our research is to explore the differentially expressed circRNAs (DEcircRNAs) and elucidate complex regulated mechanism of circRNAs in aseptic loosening. The DEcircRNAs were identified by RNA sequencing (RNA-seq) analysis. Reverse transcription-quantitative polymerase chain reaction (RT-qPCR) was adopted to corroborate these DEcircRNAs. The potential function of circRNAs in aseptic loosening tissue was identified by competing endogenous RNA (ceRNA) analysis. Enrichment analysis was performed for target mRNAs and host genes of the DEcircRNAs by Gene Oncology (GO) and Kyoto Encyclopedia of Genes and Genomes (KEGG). 257 DEcircRNAs were obtained from RNA-seq results. Following the RT-qPCR corroboration, 6 circRNAs (hsa_circ_0007482, hsa_circ_0005232, hsa_circ_0000994, hsa_circ_0000690, hsa_circ_0058092 and hsa_circ_0004496) were selected for further analysis. By circRNA-miRNA and miRNA-mRNA prediction, 6 circRNAs, 138 miRNAs and 1667 mRNAs were identified. Then, circRNA-miRNA-mRNA network was established. The result of GO and KEGG enrichment analysis suggested that the circRNAs were related with some biological functions and pathways of aseptic loosening. A novel pathogenesis and treatment strategy about aseptic loosening after THA was revealed from our study of circRNA-miRNA-mRNA network.

## Introduction

Total hip arthroplasty (THA) is one kind of successful treatment for the management of pain and function 1. One of the most common long-term complication of THA is aseptic loosening 2. Several risk factors have been proposed as possible causes 3. However, no consensus has been reached regarding the degree of influence of each one 4. An imbalance in osteogenesis and osteolysis around the joint prosthesis was recognized as the main reason of aseptic loosening, but the pathogenesis of aseptic loosening is not completely elucidated^5^.

Circular RNA (circRNA) is a special type of endogenous noncoding RNA and formed by intron or exon circularization 6. CircRNA is covalently closed loop structures with the characteristics of conservation, stabilization, enrichment, and tissue specific expression.^7^ There is growing evidence that circRNAs have multiple biological functions, including microRNA sponges, regulating target gene transcription, and forming RNA-protein complexes 7-9. Competing endogenous RNA (ceRNA) mechanism was presented as a hypothesis 10. CircRNAs can competitively bind to miRNA response elements (MREs) as natural miRNA sponges to regulate downstream mRNA expression, and take part in many human physiology and pathology through ceRNA mechanism 8, 11. While, it remain largely unknown about the roles of circRNAs in THA, especially in aseptic loosening processes.

The underlying mechanism of circRNAs in aseptic loosening after THA was investigated in our reseach. First, the RNA-seq analysis of circRNAs was used to detect the differentially expressed circRNAs (DEcircRNAs) profiles in aseptic loosening and non-aseptic loosening synovial tissue of the hip joint. Then, the DEcircRNAs were corroborated by reverse transcription-quantitative polymerase chain reaction (RT-qPCR). We predicted their target miRNAs and target mRNAs, then constructed a circRNA-miRNA-mRNA network to reveal whether DEcircRNAs acting as ceRNAs in aseptic loosening. We conducted Gene Oncology (GO) and Kyoto Encyclopedia of Genes and Genomes (KEGG) enrichment analysis on the downstream mRNAs and host genes of the DEcircRNAs to reveal the possible pathogenesis of aseptic loosening. Therefore, our finding might further elucidate the complex RNA regulatory mechanism by constructing ceRNA network and provide a novel perspective for the pathogenesis and latent diagnosis of aseptic loosening.

## Materials and Methods

### Patients

The experimental specimens were obtained from the hip joint synovial tissues of patients, who received revision operations for aseptic loosening after THA. The hip joint synovial tissues from patients with femoral neck fracture during their primary THA were acting as control specimens. 6 aseptic loosening tissues and 6 non-aseptic loosening tissues were obtained between January 2018 and November 2019 from the First Affiliated Hospital of China Medical University. 3 pairs of specimens were selected for RNA-seq, and the 6 paired specimens were used to identify the accuracy of the RNA-seq results by RT-qPCR. All specimens were quick-freezing and preserved at - 80 °C before use. Our study meet the specification of the Declaration of Helsinki, and approved by the Research Ethics Committee of the First Affiliated Hospital of China Medical University.

### Library construction and high-throughput sequencing

Total RNA from specimens for RNA sequencing was isolated by a TRIzol™ reagent (Invitrogen, CA, USA) as per the manufacturer's instructions. NanoDrop ND-1000 (Thermo Fisher Scientific, Waltham, MA, USA) was used for determinating the RNA quality and quantity. Denaturing agarose gel electrophoresis was used for testing the RNA integrity and gDNA contamination. The remnant RNA was preserved at - 80 °C for later use.

Approximately 1-2 μg total RNA from each specimens was used for library construction. Extracted mRNA was enriched with NEB Next® Poly (A) mRNA Magnetic Isolation Module (New England Biolabs). KAPA Stranded RNA-Seq Library Prep Kit (Illumina) was used for the library construction. RNA-seq libraries were subjected to quality control analysis by Agilent 2100 Bioanalyzer. Libraries were quantified by qRT-PCR. Samples were sequenced using Illumina X-ten/NovaSeq. The RNA-seq data were deposited in GEO (Accession code: GSE149315).

### Data analysis

Illumina X-ten/NovaSeq produced raw sequencing data in FASTQ format. The raw sequencing data were submitted to sequencing quality control by FastQC to assess whether to be used for subsequent data analysis. The trimmed data generated by the pre-processing and filtering steps were mapped to the reference genome. First, StringTie software was used to compare the results to the known transcriptome and calculate the transcriptional abundance. Then, Ballgown was used for quantification of gene expression levels, as well as analysis of intergroup differential expression of genes. The differentially expressed genes were screened with a width of the difference ≥1.5 times, a P-value ≤ 0.05 and an internal FPKM (Fragments Per Kilobaseof gene/transcript model per Million mapped fragments) mean value ≥0.5. Backsplice junction reads count of circRNA was quantified by STAR software through mapping to the reference genome, and its backsplice junction reads detection and reads count statistics were done by the CIRCexplorer2 software. EdgeR package was used for determining the significantly DEcircRNAs with the filter criteria of P-values <0.05 and |log2FC| ≥ 0.585.

### RT-qPCR

We selected six aseptic loosening synovial tissues, matched with 6 control samples. Total RNA from specimens was isolated by a TRIzol™ reagent (Invitrogen, CA, USA) following the manufacturer`s instruction. To quantify the amount of each circRNA, cDNA was synthesized by reverse transcription using SuperScript™ III Reverse Transcriptase (Invitrogen) on Gene Amp PCR System 9700 (Applied Biosystems) from 1 μg of total RNA. Subsequently, ViiA 7 Real-time PCR System (Applied Biosystems) was used for qRT-PCR by 2X PCR master mix (Arraystar) following the manufacturer`s instruction. The relative expression of each circRNA was determined by 2^-ΔCT^ method. GAPDH was used as internal control. Primer 5.0 software was used for the design of primer sequences in our study. Paired T-test was adopted for analysis of significance between experimental and control group by SPSS 22.0. And P-value < 0.01 indicates a statistical significance.

### Construction of the ceRNA network

CeRNA hypothesis that the circRNA shared the same miRNA with mRNA was acted as the theoretical basis for network construction. MREs were the miRNA binding sites which were foundation of ceRNA hypothesis, and crosstalk of RNA transcripts can be caused by competing for common miRNAs with same MREs. Home-made miRNA target prediction software based on TargetScan (http://www.targetscan.org/vert_72/) 12 and miRanda (http://www.microrna.org/microrna/) 13 was used for prediction of circRNA-miRNA and miRNA-mRNA interaction. The energy score of miRanda should be smaller than -10 and the structure score should be higher than 140. The context+ score should be smaller than -0.05. As we have already enrolled the corroborated DEcircRNAs, cytoscape software (https://cytoscape.org/index.html) 14 was used for the circRNA-miRNA-mRNA network construction based on their target miRNAs and mRNAs.

### GO and KEGG enrichment analysis

Database for Annotation, Visualization and Integrated Discovery (DAVID) (http://david.abcc.ncifcrf.gov/) 15 was conducted for functional enrichment analysis to understand the underlying mechanism of these DEcircRNAs and ceRNA network. Then the target mRNAs and host genes of DEcircRNAs were subjected to enrichment analysis by GO annotation and KEGG pathway to reveal the possible functional classification. GO enrichment analysis elucidated the functional annotations including biological process (BP), cellular component (CC), and molecular function (MF). KEGG enrichment analysis was conducted to elucidate the target genes related signaling pathways.

## Results

### Identification of differentially expressed circRNAs and genes

**Figure 1:**
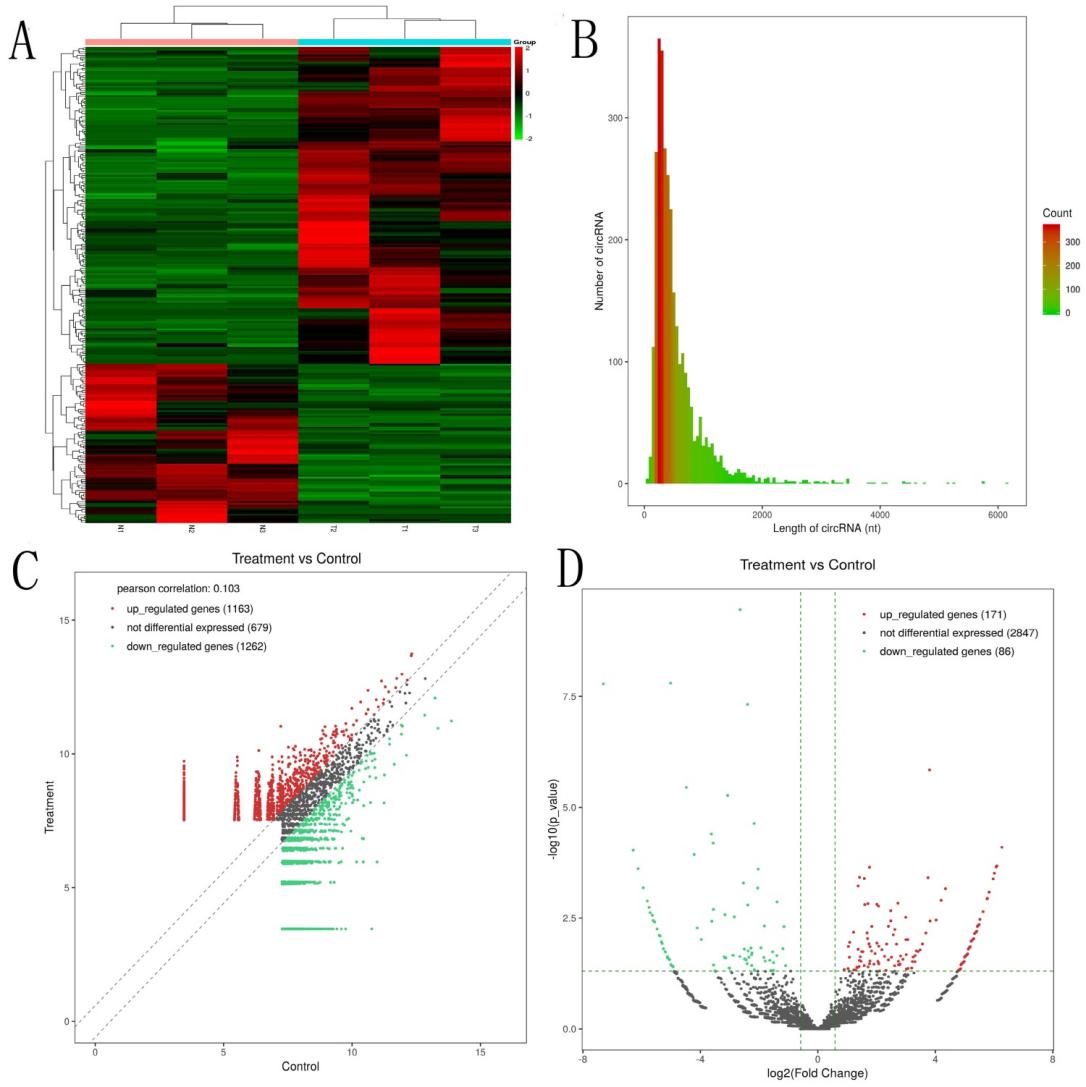
** Identification of circular RNAs by RNA-seq analyses in synovial tissues of the hip joint. A.** Clustered heat map of the differentially expressed circRNAs in three paired aseptic loosening and non-aseptic loosening synovial tissues. Rows represent circRNAs while columns represent tissues. **B.** The length distribution of exonic circRNAs.** C.** Scatter plot showed the distributions of circRNAs in more direct way.** D.** volcano plot showed the significantly differentially expressed circRNAs.

**Figure 2:**
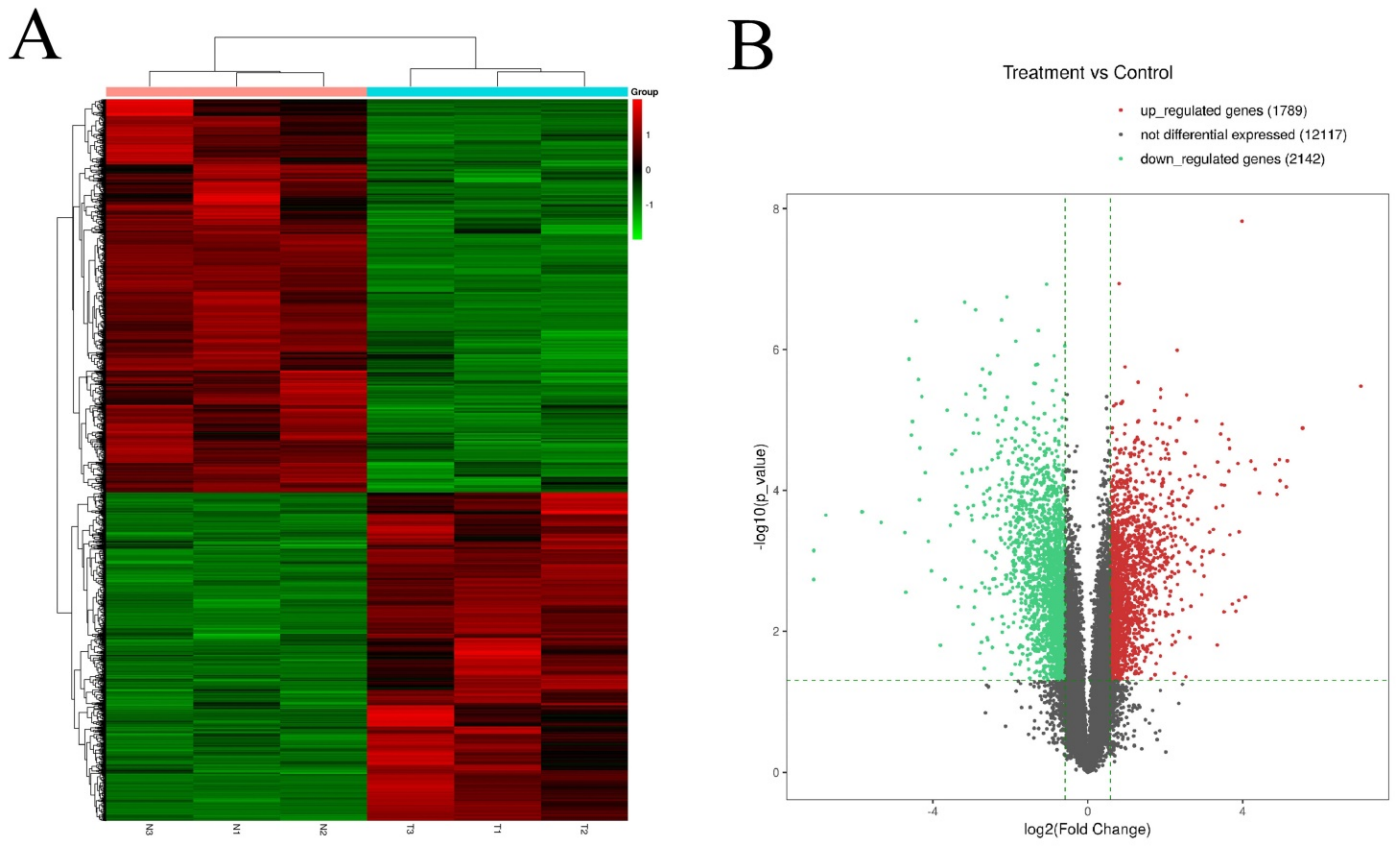
** Identification of differentially expressed genes by RNA-seq analyses in synovial tissues of the hip joint. A.** Clustered heat map of the differentially expressed genes in three paired aseptic loosening and non-aseptic loosening synovial tissues. Rows represent genes while columns represent tissues. **B.** volcano plot showed the significantly differentially expressed genes.

The expression profiles of circRNAs are exhibited in heatmap (Fig. 1A). The length of most circRNAs was less than 2000 nucleotide (nt) (Fig. 1B). The different circRNAs expression profiles are exhibited in scatter plot (Fig. 1C). Overall, 257 significantly DEcircRNAs (|log2FC| ≥ 0.585 and P < 0.05) were discovered and are exhibited in volcano plot (Fig. 1D). And, 171 circRNAs were upregulated and 86 circRNAs were downregulated in aseptic loosening tissues. Among the 257 significantly DEcircRNAs, 243 circRNAs (94.55%) have been validated in other studies and included in circBase, and the other 14 (5.45%) are novel. Furthermore, these DEcircRNAs were transformed from 237 host genes (158 upregulated and 79 downregulated). The expression profiles of genes are exhibited in heatmap (Fig. 2A). Overall, 1789 genes were significantly upregulated and 2142 genes were significantly downregulated (≥1.5 times difference, P-value ≤ 0.05 and FPKM≥0.5) (Fig. 2B). Through comparison, 34.2% (54/158) host genes of upregulated circRNAs was upregulated, and 44.3% (35/79) host genes of downregulated circRNAs was downregulated.

### Validation of identified circRNAs by RT-qPCR

**Figure 3:**
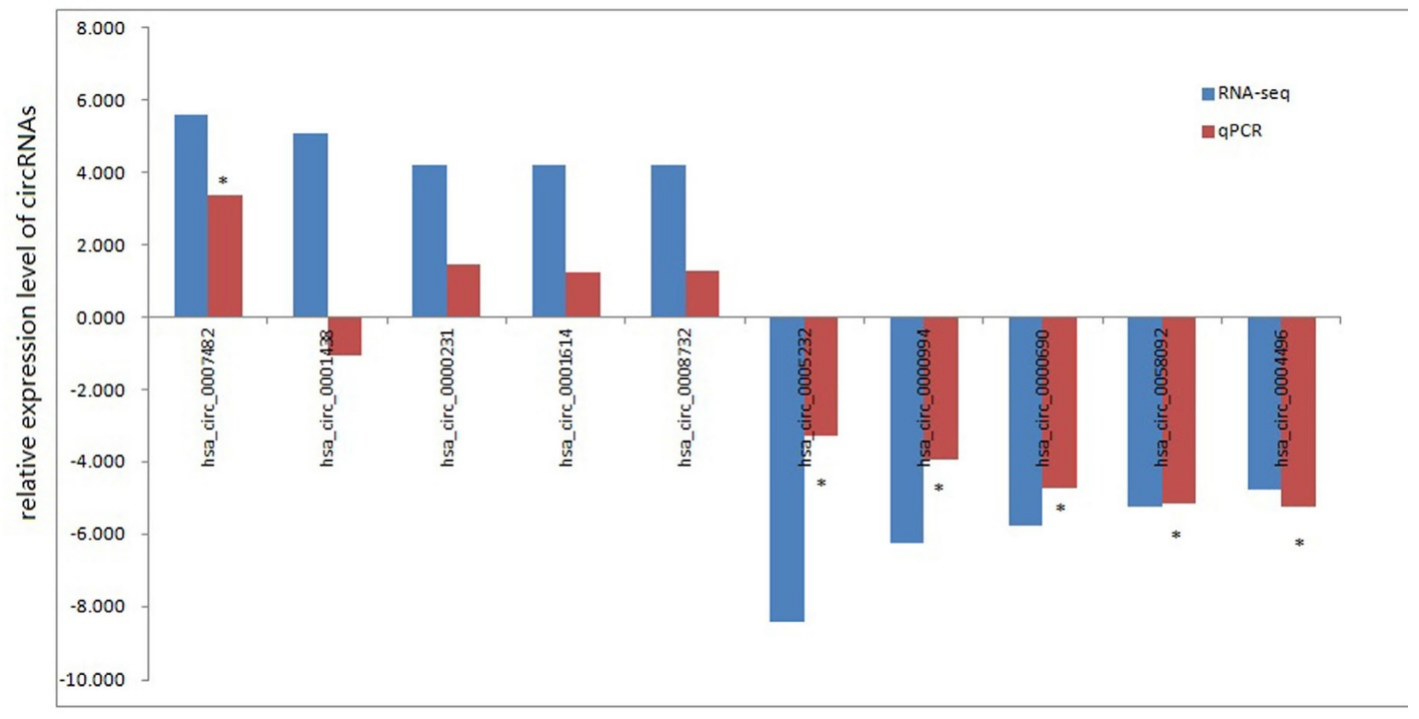
** RNA-seq and qRT-PCR analysis of selected circRNAs.** *, *P* < 0.01 in qRT-PCR verification when comparing data between aseptic loosening and control groups.

To test whether the DEcircRNAs discovered through the RNA-seq results were valid, we selected 5 upregulated and 5 downregulated circRNAs for validation through RT-qPCR. We used the following criteria for circRNA selection: (1) included in circBase; (2) |log2FC| >2; (3) p-value <0.05; (4) exonic-related circRNAs; and (5) CPM value was not zero. RT-qPCR was performed in 6 pairs aseptic loosening and non- aseptic loosening tissues to validate the expression of the 10 DEcircRNAs. Nine of ten selected DEcircRNAs were the same change tendency though the data from RNA-seq and RT-qPCR. Among them, 1 upregulated circRNA (hsa_circ_0007482) and 5 downregulated circRNAs (hsa_circ_0005232, hsa_circ_0000994, hsa_circ_0000690, hsa_circ_0058092 and hsa_circ_0004496) were statistically significant and these 6 circRNAs were candidates for further study (Fig. 3). The RT-qPCR results were in accord with our circRNA-seq results, so our circRNA-seq profiles are high reliability.

### Biological function analysis of host genes

**Figure 4:**
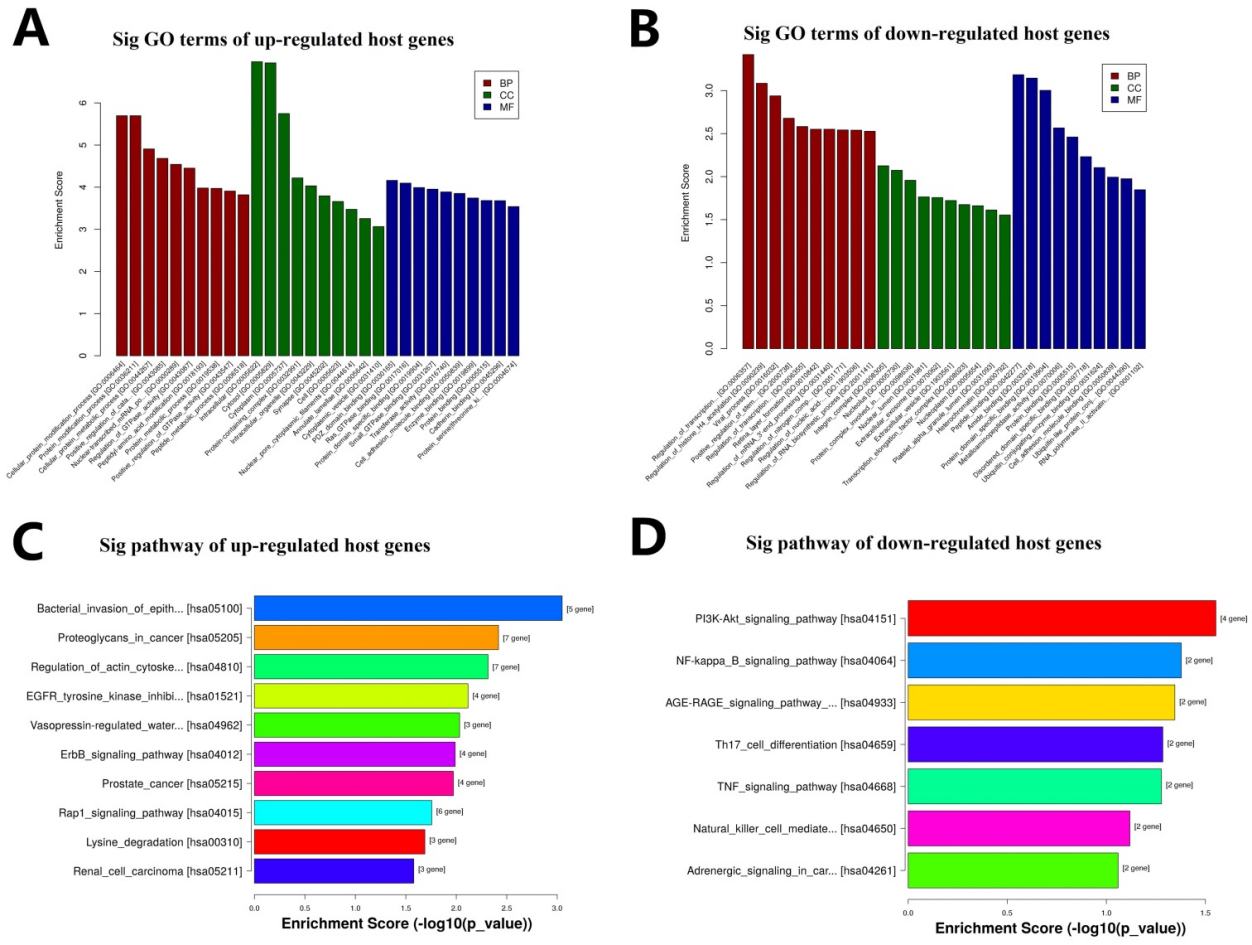
** GO and KEGG analysis of DEcircRNAs host genes. A.** GO annotation for host genes of these up-regulated circRNAs under the theme of BP, CC and MF. **B.** GO annotation for host genes of these down-regulated circRNAs under the theme of BP, CC and MF. C. KEGG enrichment analysis for host genes of these up-regulated circRNAs. D. KEGG enrichment analysis for host genes of these down-regulated circRNAs.

The biological function of circRNAs would be related to the known function of their host linear transcripts because of circRNAs are transformed from their host linear RNAs. GO and KEGG enrichment analysis was used for the 237 host genes of these 257 DEcircRNAs. The enrichment analysis result of GO annotation for host genes of these upregulated circRNAs is listed in Fig. 4A, and the result of GO annotation for host genes of these downregulated circRNAs is listed in Fig. 4B, including BP, CC, and MF. The enrichment analysis result of KEGG pathway show that the host genes of these upregulated circRNAs were most significantly enriched in Rap1 signaling pathway, ErbB signaling pathway, bacterial invasion of epithelial cells, proteoglycans in cancer, regulation of actin cytoskeleton, EGFR tyrosine kinase inhibitor resistance, vasopressin-regulated water reabsorption, prostate cancer, lysine degradation, and renal cell carcinoma (Fig. 4C). The enrichment analysis result of KEGG pathway show that the host genes of these downregulated circRNAs were most significantly enriched in PI3K-Akt signaling pathway, NF-kappa B signaling pathway, AGE-RAGE signaling pathway in diabetic complications, Th17 cell differentiation, TNF signaling pathway, natural killer cell mediated cytotoxicity, and adrenergic signaling in cardiomyocytes (Fig. 4D).

### CircRNA-miRNA-mRNA network

**Figure 5:**
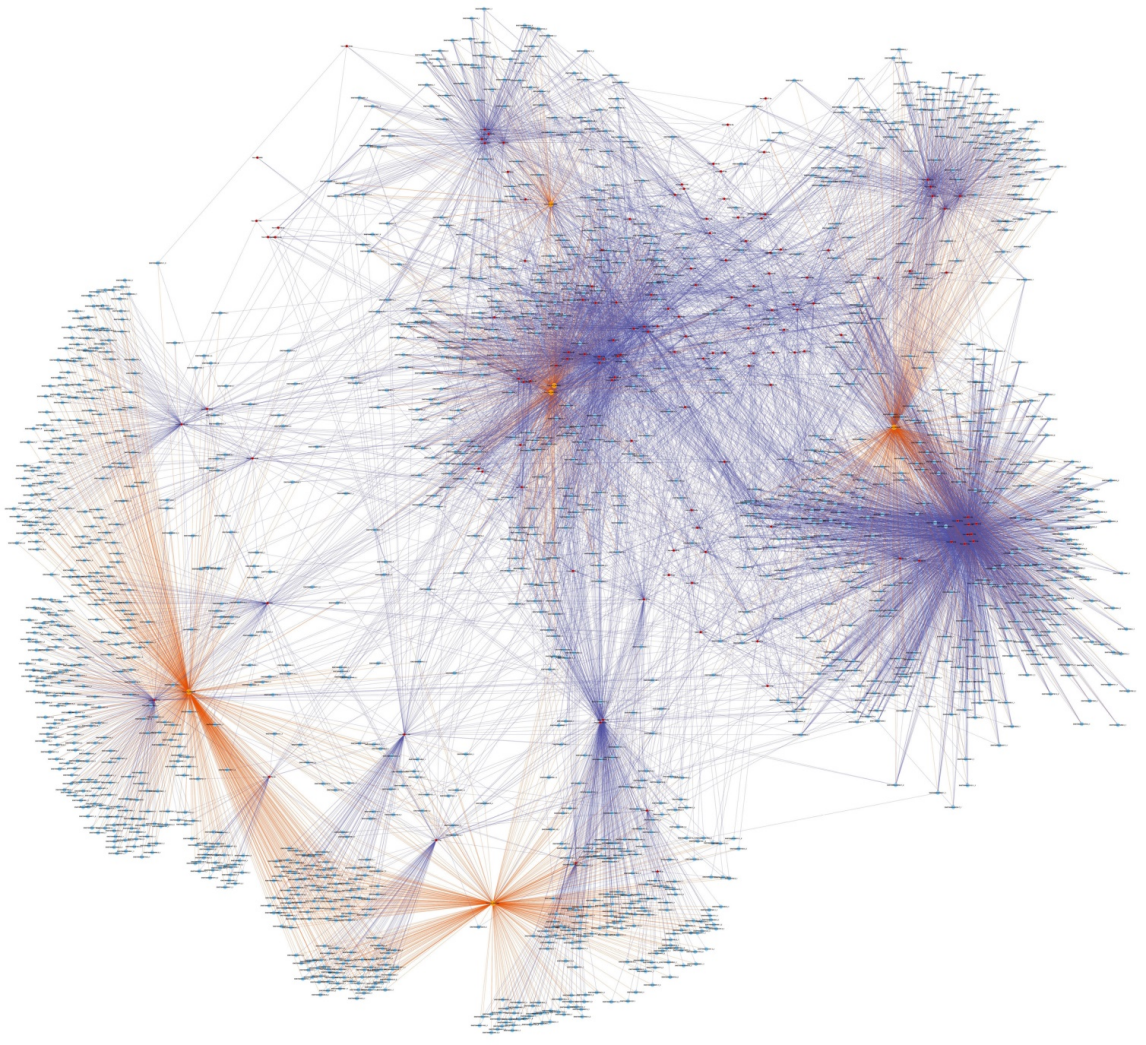
** CircRNA-miRNA-mRNA network containing the high score interactions.** The yellow circle represents circRNAs, the red circles represent miRNAs, and the blue circles represent mRNAs.

**Figure 6:**
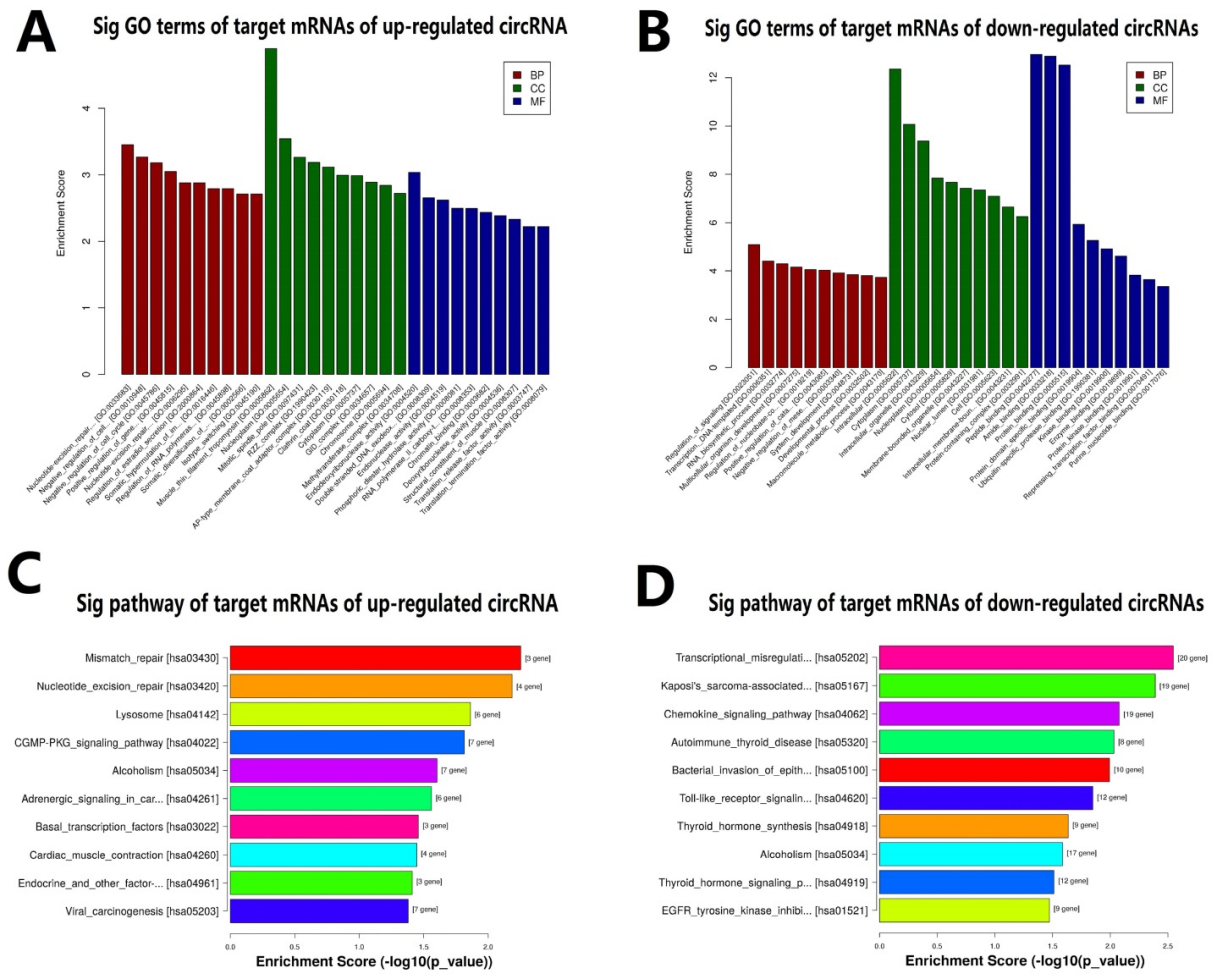
** GO and KEGG analysis of target genes. A.** GO annotation for target mRNAs of up-regulated circRNA (hsa_circ_0007482) under the theme of BP, CC and MF. **B.** GO annotation for target mRNAs of these down-regulated circRNAs (hsa_circ_0005232, hsa_circ_0000994, hsa_circ_0000690, hsa_circ_0058092, and hsa_circ_0004496) under the theme of BP, CC and MF. **C.** KEGG enrichment analysis for target mRNAs of these up-regulated circRNA (hsa_circ_0007482). **D.** KEGG enrichment analysis for target mRNAs of these down-regulated circRNAs (hsa_circ_0005232, hsa_circ_0000994, hsa_circ_0000690, hsa_circ_0058092, and hsa_circ_0004496).

We were the first to establish a ceRNA network in the aseptic loosening after THA according to ceRNA mechanism that ceRNA members effectively compete the same MREs to affect one another transcripts. Through our RNA-seq and qRT-PCR results, hsa_circ_0007482, hsa_circ_0005232, hsa_circ_0000994, hsa_circ_0000690, hsa_circ_0058092, and hsa_circ_0004496 were selected for further analysis. Their potential target miRNAs and mRNAs were predicted. A total of 138 circRNA-miRNA interactions contain 6 circRNAs and 138 miRNAs were identified. Total 1667 target mRNAs of the aforementioned 138 miRNAs were obtained. We created a circRNA-miRNA-mRNA network by integrating the circRNA-miRNA interactions and miRNA-mRNA interactions using Cytoscape (Fig. 5). GO and KEGG enrichment analysis was used for the target mRNAs of these 6 DEcircRNAs. The enrichment analysis result of GO annotation for target mRNAs of upregulated circRNA (hsa_circ_0007482) is listed in Fig. 6A , and the result of GO annotation for target mRNAs of downregulated circRNAs (hsa_circ_0005232, hsa_circ_0000994, hsa_circ_0000690, hsa_circ_0058092, and hsa_circ_0004496) is listed in Fig. 6B. The enrichment analysis result of KEGG pathway show that the target mRNAs of upregulated circRNA (hsa_circ_0007482) were most significantly enriched in mismatch repair, nucleotide excision repair, lysosome, cGMP-PKG signaling pathway, alcoholism, adrenergic signaling in cardiomyocytes, basal transcription factors, cardiac muscle contraction, endocrine and other factor-regulated calcium reabsorption, and viral carcinogenesis (Fig. 6C). The enrichment analysis result of KEGG pathway show that the target mRNAs of downregulated circRNAs (hsa_circ_0005232, hsa_circ_0000994, hsa_circ_0000690, hsa_circ_0058092, and hsa_circ_0004496) were most significantly enriched in Toll-like receptor signaling pathway, transcriptional misregulation in cancer, Kaposi's sarcoma-associated herpesvirus infection, chemokine signaling pathway, autoimmune thyroid disease, bacterial invasion of epithelial cells, thyroid hormone synthesis, alcoholism, thyroid hormone signaling pathway, and EGFR tyrosine kinase inhibitor resistance (Fig. 6D). The ceRNA network that we constucted may supply a new viewpoint for the pathogenesis and treatment of aseptic loosening.

## Discussion

CircRNA was firstly identified in 1976, and was gradually found in viruses, viroids, and tetrahymena, while circRNAs were initially considered as a result of dysregulation or abnormal RNA splicing 16. Due to the restriction of traditional RNA detection methods, circRNAs were neglected for a long periods. Hsu and Coca-Prados firstly reported the phenomenon of the RNA presenting itself as a circle in the eukaryotic cytoplasm by electron microscopic evidence 17. In recent years, a mass of circRNAs have been successfully found in various tissues and species with the progresses of high-throughput sequencing technology and bioinformatics analysis 18. CircRNAs expressions are extraordinary abundant compared with their linear RNA isoforms from the same genes 19. CircRNAs were thought to be new star RNAs due to recent discovery of their diverse functions, vast abundance, and frequent tissue-specific expression, and played important roles in various cancers 19. Further studies indicated that some circRNAs also played a important role in various types of non-cancer diseases and provided potential implications in accurate diagnosis and therapies 11, 20. While, only a few studies on the role of circRNAs in orthopedic disorders have been reported, and the contributions of circRNAs to pathogenesis of aseptic loosening remain basically unknown. In our research, we first elucidated circRNA expression profile in human aseptic loosening synovial tissue of the hip joint by high-throughput sequencing. Our results indicated that circRNA was significantly different expression from aseptic loosening and non-aseptic loosening tissue. Our findings strongly suggested different expression patterns of circRNA in aseptic loosening tissues played an important role in pathogenesis.

Now it is widely accepted that circRNAs have a variety of regulatory mechanisms to exert their biological function. One major mechanism is regulating the splicing or transcription of their host genes by interacting with RNA binding proteins 11. Most studies show that the circRNAs expression level was largely related with their linear counterpart from the same host gene 21, 22. Based on this, GO and KEGG enrichment analysis was used to predict the putative functions of the host genes of the DEcircRNAs. The KEGG pathway analysis indicated that the host genes were enrichment in some important signaling pathways. Rap1 signaling pathway was related with the host genes of upregulated circRNAs. PI3K-Akt signaling pathway and TNF signaling pathway were related with the host genes of downregulated circRNAs. Previous study had reported that Rap1 signaling pathway had been reported to contribute to osteoblast proliferation and differentiation in osteoporosis 23. Also, the PI3K/AKT pathway participated in the regulation of osteoclastogenesis by wear particles during aseptic loosening 24. In addition to regulating osteoclast differentiation, PI3K/AKT pathway was also involved in osteoblast formation 25. Recent investigation had indicated that TNF-α could mediate autophagy and apoptosis of osteoblast 26. Autophagy was recognized as an important process for aseptic loosening 27. The presence of wear particles in the main cell types of synovial tissues triggered autophagy, then osteoblast and osteoclast differentiation was sequentially regulated by autophagy and led to periprosthetic aseptic loosening 28, 29.

Study had shown that major circRNAs expression profiles followed their host gene expression profiles 30. Our analysis of the host genes may show that these DEcircRNAs were also correlated with these signalling pathways, but further verification is required.

Another key feature of circRNAs is serving as a sponge to miRNAs and exerts function as ceRNAs to control gene expression. We predicted the interactive miRNAs of validated 6 DEcircRNAs, and predicted the interactive downstream target mRNAs of aforementioned miRNAs. We constructed circRNA-miRNA-mRNA network by cytoscape. Then GO and KEGG enrichment analysis was conducted to predict the functions of downstream target mRNAs. The KEGG pathway analysis indicated that the target mRNAs of upregulated circRNA (hsa_circ_0007482) were enrichment in cGMP-PKG signaling pathway, and the target mRNAs of downregulated circRNAs (hsa_circ_0005232, hsa_circ_0000994, hsa_circ_0000690, hsa_circ_0058092, and hsa_circ_0004496) were enrichment in chemokine signaling pathway and Toll-like receptor signaling pathway. According to current study, activation of cGMP-PKG pathway could promote rat osteoblast differentiation and maturation 31. And study showed that wear particles activated resident cells of synovial tissue to release various regulatory molecules and inflammatory mediators, among them chemokines were the most prominent 32. The chemokine system played an important role in the inflammatory and osteolysis processes leading to periprosthetic aseptic loosening 33. Toll like receptors were found in the tissues around aseptic loosening implants, it had been speculated to play a role in hip implant aseptic loosening 34. Previous study showed that Toll like receptors-2 and -4 regulated responses play an important role in the progresses of PE wear debris-induced osteolysis in the murine calvarium model 35. We were the first to establish a ceRNA network in the aseptic loosening after THA, and it will provide novel information for exploring the role of circRNAs in the therapeutic targets and pathogenesis of aseptic loosening process. Further studies are required to verify the enormous potential role of ceRNA in circRNA-miRNA-mRNA axis in aseptic loosening.

In general, 171 circRNAs were upregulated and 86 circRNAs were downregulated in aseptic loosening synovial tissues compared to non-aseptic loosening synovial tissues. GO and KEGG analysis of the DEcircRNAs host genes shows that PI3K-Akt signaling pathway, Rap1 signaling pathway, and autophagy are the most common terms. CeRNA network was constructed based on the 6 validated DEcircRNAs. Enrichment analysis of the target genes of these 6 validated DEcircRNAs shows that the target genes mainly took part in the Toll-like receptor signaling pathway and chemokine signaling pathway. The signaling pathways mentioned above are closely related to osteolysis. Therefore, the ceRNA network may reveal novel mechanisms for aseptic loosening, and circRNAs may serve as candidates in aseptic loosening. The functions of DEcircRNAs from our RNA-seq data warrant further investigations.

## Abbreviations

CircRNA: circular RNA
THA: total hip arthroplasty
DEcircRNAs: differentially expressed circRNAs
RNA-seq: RNA sequencing
RT-qPCR: reverse transcription-quantitative polymerase chain reaction
ceRNA: competing endogenous RNA
MREs: miRNA response elements
GO: Gene Oncology
KEGG: Kyoto Encyclopedia of Genes and Genomes
